# Increased Serum Levels of Inflammatory Mediators and Low Frequency of Regulatory T Cells in the Peripheral Blood of Preeclamptic Mexican Women

**DOI:** 10.1155/2014/413249

**Published:** 2014-12-07

**Authors:** Mario Adan Moreno-Eutimio, José María Tovar-Rodríguez, Karina Vargas-Avila, Nayeli Goreti Nieto-Velázquez, María Guadalupe Frías-De-León, Mónica Sierra-Martinez, Gustavo Acosta-Altamirano

**Affiliations:** ^1^Immunobiology Laboratory, Hospital Juárez de México, Ministry of Health, 07760 Mexico City, Mexico; ^2^Research Directorate, Hospital Juárez de México, Ministry of Health, 07760 Mexico City, Mexico; ^3^Molecular Genetics Laboratory, Hospital Juárez de México, Ministry of Health, 07760 Mexico City, Mexico

## Abstract

Regulatory T cells (T_regs_; CD4+CD25^high^Foxp3+) are critical in maintaining immune tolerance during pregnancy and uterine vascularization. In this study, we show that, in Mexican women with different preeclamptic severity levels, the number of T_regs_ and the subset of CD4+CD25^high^Foxp3+ are decreased compared with those of normotensive pregnant women (NP). Moreover, a systemic inflammatory state is a pivotal feature in the pathogenesis of this disorder and could be related to hypertension and endothelial dysfunction. Likewise, we observed elevated levels of IL-6, TNF-*α*, and IL-8 in the serum of severe preeclamptic patients (SPE); no differences were found in the IL-1*β* and IL-10 levels compared with those of NP patients. An analysis of chemokines in the preeclamptic serum samples showed high levels of CXCL10, CCL2, and CXCL9. Our findings suggest that the preeclamptic state is linked with systemic inflammation and reduced numbers of T_regs_.

## 1. Introduction

Preeclampsia (PE) is a complication of pregnancy that is characterized by hypertension, proteinuria, and maternal systemic inflammation after 20 weeks of gestation, when innate and adaptive responses play roles in the pathogenesis of this disorder [1]. PE is an important cause of maternal and fetal morbidity and mortality worldwide. In developing countries, such as Mexico, 34% of maternal deaths are linked to PE, which is also the major cause of premature delivery [2]. Several factors, such as first pregnancy, a maternal age >40, hypertension, obesity, and single nucleotide polymorphisms in several genes, including those involved in angiotensin activity and oxidative stress, have been associated with PE development [1, 3–6].

Although the pathogenesis of PE is not fully understood, several works have linked inflammation to cardinal features of this disorder. For instance, preeclamptic placenta secretes several inflammatory molecules as a result of the hypoxic state developed from a lack of vessel remodeling in the uterus [7–9].

It is well known that regulatory T cells (T_regs_) are a particular subset of T lymphocytes (CD4+, CD25^high^, Foxp3+) that maintain immunological self-tolerance, suppress the inflammatory state, and induce immune homoeostasis [10, 11]. During pregnancy, T_regs_ are able to maintain immune tolerance by suppressing natural killer (NK) cells and T cell responses against allogeneic paternal antigens and self-antigens involved in rejection and labor complications such as PE [8, 12]. Additionally, a number of findings have indicated that the increased expression of inflammatory mediators, such as cytokines and chemokines, could be potential mediators of endothelial dysfunction in preeclamptic patients [13]. A normal pregnancy is characterized by a shift toward Th2-type immunity and the inhibition of cytotoxic Th1 immune responses [8].

Several soluble factors, such as chemokines and cytokines, play key roles during inflammation in PE. For instance, chemokines, which are chemotactic cytokines, participate in several biological processes, such as cellular lymphoid organogenesis, angiogenesis, and adhesion molecules expression [14]. In addition to the secretion of inflammatory cytokines such as IL-6 and TNF-*α*, the amount of circulating chemokines is elevated in PE [15–17].

Several studies have been published describing that normal human pregnancy is associated with elevated numbers of T_regs_ cells [18]. Whether deficiencies in the number or function of T_regs_ cells are implicated in the development of preeclampsia remains controversial. Recent reports described decreased numbers of T_regs_ cells in preeclampsia compared with normal pregnancy [19–24], whereas others found comparable frequencies [25, 26]. Furthermore, most of these studies used CD25 as a T_regs_ cell marker. However surface markers such as CD25 are also dynamically expressed on the surface of newly activated CD4+ T-helper cells. Using the intracellular marker Foxp3 is therefore superior and more accurate. Moreover, numerous studies have indicated that ethnicity may be associated with the risk of preeclampsia [27]. A recent study has shown that Mexican women have the highest risk to develop preeclampsia [28].

The purpose of the present study was to evaluate the number of T_regs_ in Mexican women with PE and evaluate their correlation with the circulating chemokines and cytokines.

## 2. Material and Methods

### 2.1. Patients and Healthy Donors

Forty-nine moderated preeclamptic pregnant (MPE), 24 severe preeclamptic pregnant (SPE), 51 normotensive pregnant (NP), and 27 nonpregnant healthy control (HC) women were studied. The study participants were enrolled in the Department of Obstetrics and Gynecology of the Hospital Juárez de México. Ethical approval for the study was obtained from the Hospital Juárez de México Ethics Committee. All women were informed of the goal of the study, and informed consent was obtained from all patients. The MPE group comprised women matching the diagnostic criteria of the International Society for the Study of Hypertension in Pregnancy (ISSHP) [29]: two blood pressure readings of ≥140/90 mmHg were taken at least with >300 mg proteinuria over 24 hours or +1 on dipstick analysis. The SPE group comprised women matching the diagnostic criteria of the ISSHP: two blood pressure readings of ≥160/110 mmHg were taken at least with >5 g proteinuria over 24 hours or +3 on dipstick analysis. None of the patients had HELLP syndrome or eclampsia. The exclusion criteria for this group included concurrent medical problems that may result in disordered inflammatory responses, such as diabetes, autoimmune diseases, and the spontaneous rupture of membranes. Fifty-one maternal blood samples were drawn from normotensive pregnant women who all delivered healthy babies. These were matched by gestational age to the moderated preeclamptic pregnant group at the time of blood sampling. All pregnancies were singleton.

Clinical characteristics of patients with preeclampsia, normotensive pregnant women, and nonpregnant healthy women are summarized in Table 1.

### 2.2. Blood Sample Collection

Four mL of whole blood were collected from the antecubital vein of each subject. These samples were centrifuged (Heraeus Megafuge 40R, Thermo Fisher Scientific Inc., MA, USA) at 2500 rpm for 15 min to isolate the supernatant, which was frozen at −70°C until analysis of the cytokine and chemokine profiles.

### 2.3. Isolation of Peripheral Blood Mononuclear Cells

Peripheral blood samples from all women were collected into EDTA tubes (Becton Dickinson and Company, Franklin Lakes, NJ, USA). All of the blood samples were processed within 2 h after sampling. Peripheral blood mononuclear cells (PBMC) were isolated using the density centrifugation technique (Ficoll-Paque PLUS, Amersham Biosciences, Uppsala, SE) and then immediately utilized.

### 2.4. Flow Cytometric Analysis of T_regs_

To identify T_regs_, PBMC were stained both for surface antigens with a FITC-conjugated mAb specific for CD4 and a PE-conjugated mAb specific for CD25 and for intracellular molecules with an APC-conjugated mAb specific for Foxp3 (eBioscience, San Diego, CA, USA) in accordance with the manufacturer's instructions. Briefly, 1 × 10^6^ PBMC were stained with fluorochrome-conjugated mAbs specific for cell surface antigen markers for 20 min in the dark at 4°C. After the initial staining, the cells were washed twice using phosphate buffered saline (PBS) at pH 7.4, followed by surface marker fixation. To stain for intracellular Foxp3, cells were first permeabilized with a permeabilization/fixation buffer (eBioscience, San Diego, CA, USA) and then stained using the anti-Foxp3 mAb. The negative control samples were incubated with isotype-matched antibodies. After incubation, the cells were resuspended in 200 *μ*L of PBS for subsequent flow cytometry analysis using an Accuri C6 flow cytometer (BD Biosciences, San Jose, CA, USA). The resultant data were analyzed using the FlowJo software V10 (Tree Star, San Carlos, CA, USA).

Lymphocytes were gated based on both forward and side scatter parameters, FSC and SSC, respectively. After additional gating for CD4+ cells, the proportions of CD4+CD25+, CD4+Foxp3+, and CD4+CD25^high^Foxp3+ cells were determined.

### 2.5. Cytokines and Chemokines

Serum samples obtained from patients were analyzed for cytokine and chemokine levels using a BD cytometric bead array (CBA) human chemokine kit and a human inflammatory cytokine kit (BD Biosciences, San Jose, CA) according to the manufacturers' instructions. Capture beads were first added to the serum sample, followed by the PE detection reagent. The samples were incubated for 3 h at room temperature, washed with the assay wash buffer, and suspended again in wash buffer. CBAs were then run on an Accuri C6 flow cytometer and analyzed using the FCAP Array v3.0.1 software (Soft Flow Hungary Ltd, Pécs, Hungary).

### 2.6. Statistical Analysis

Normality was determined using Shapiro-Wilk test. The significance of the difference between groups was analyzed with one-way ANOVA test with Bonferroni correction. Analyses were performed using GraphPad Prism v5.0 (GraphPad Software Inc., San Diego, CA, USA).

## 3. Results

### 3.1. Decreased Percentage of T_regs_ Was Found in Preeclamptic Patients with Different Severity Levels of the Disorder

**Figure 1:**
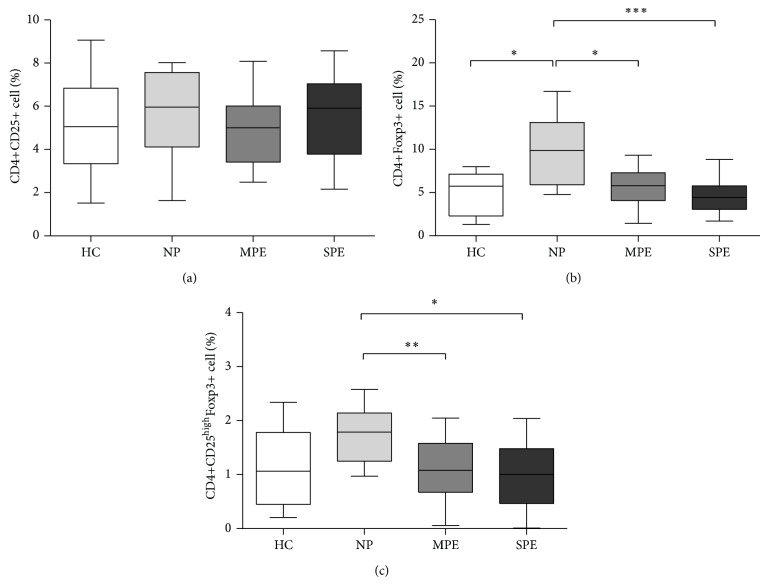
T_regs_ frequency is reduced in preeclamptic patients. Comparison of the frequency of CD4+CD25+ cells (a), CD4+Foxp3+ cells (b), and CD4+CD25^high^Foxp3+ cells (c) in peripheral maternal blood (levels as percentage of CD4+ cells) in healthy pregnant controls and preeclamptic patients. HC: healthy control; NP: normotensive pregnant; MPE: moderated preeclamptic; SPE: severe preeclamptic (^*^ *P* < 0.05, ^**^ *P* < 0.01, and ^***^ *P* < 0.001).

To evaluate the presence of T cells in healthy pregnant patients and preeclamptic patients with MPE and SPE, we characterized the percentage of CD4+CD25+ cells that represent the total of CD4+-activated T cells plus T_regs_ populations (Figure 1(a)). Our results showed no differences between the groups with PE and both the HC and NP.

Furthermore, CD4+Foxp3+ cells significantly decreased in women with MPE (*P* < 0.05) and SPE (*P* < 0.001) in comparison with NP. The NP group also presented an elevated percentage of CD4+Foxp3+ (*P* < 0.05) compared with HC (Figure 1(b)).

In addition to CD4+Foxp3+, we evaluated the percentage of CD4+CD25^high^Foxp3+ cells (Figure 1(c)), which express high levels of CD25 and are related to potent suppressor activity mediated by cell contact. Our findings show that MPE (*P* < 0.01) and SPE (*P* < 0.05) patients had lower percentages of both CD4+CD25^high^Foxp3+ and CD4+Foxp3+ cells. These data, and as was described in several works, indicate that different T_regs_ subsets are decreased in preeclamptic patients. The number of CD4+CD25^high^Foxp3+ cells represent 1-2% of the total CD4+ circulating cells, which is the most potent suppressor subset of T_regs_ for the maintenance of immune tolerance and homoeostasis, as related to hypertension during pregnancy.

### 3.2. Preeclampsia Is Associated with a Higher Circulating Concentration of Inflammatory Cytokines

**Figure 2:**
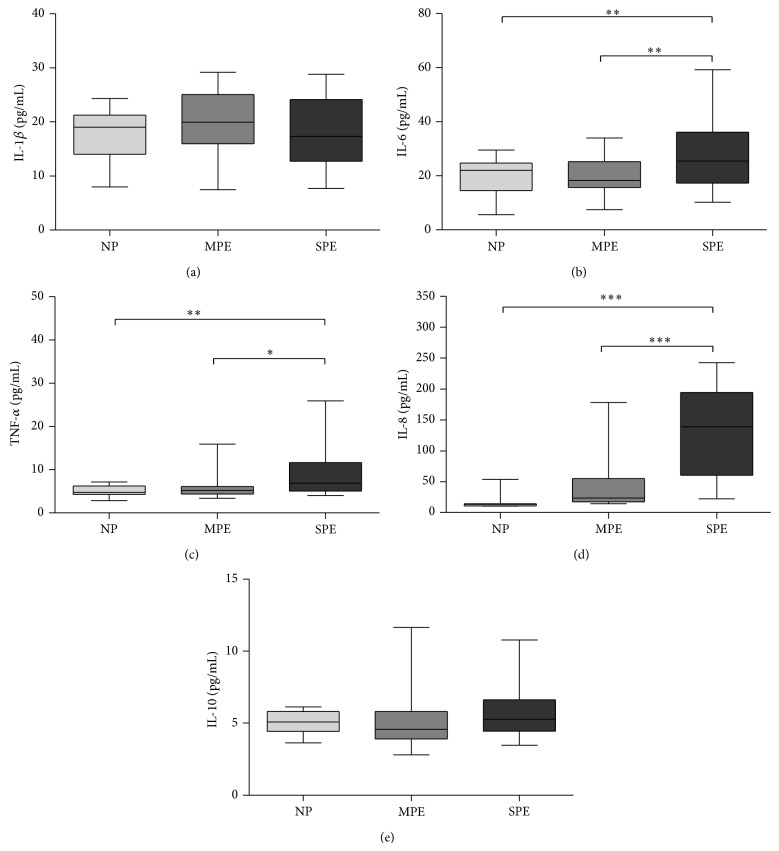
Inflammatory cytokines are deregulated in SPE patients. Plasma levels of inflammatory mediators are elevated in preeclamptic women. Plasma was obtained from normotensive pregnant (*n* = 51), moderated preeclamptic pregnant (*n* = 49), and severe preeclamptic pregnant (*n* = 24) women and inflammatory markers were analyzed by CBA microarrays (^*^ *P* < 0.05, ^**^ *P* < 0.01, and ^***^ *P* < 0.001).

In patients at different stages of PE, we measured the circulating levels of inflammatory cytokines that have been linked with complications in this disorder. In SPE patients, our results show that the level of inflammatory cytokines, such as IL-6, TNF-*α*, and IL-8, were higher than those in NP (*P* < 0.01, *P* < 0.01, and *P* < 0.001, resp.) and MPE (*P* < 0.01, *P* < 0.05, and *P* < 0.001, resp., Figures 2(b)–2(d)).

Interestingly, the concentrations of IL-1*β* and IL-10 across the different groups of patients were approximately the same (Figures 2(a) and 2(e)).

### 3.3. Chemokines Related to Antiangiogenic Properties and Inflammatory States Are Increased in Preeclamptic Patients

**Figure 3:**
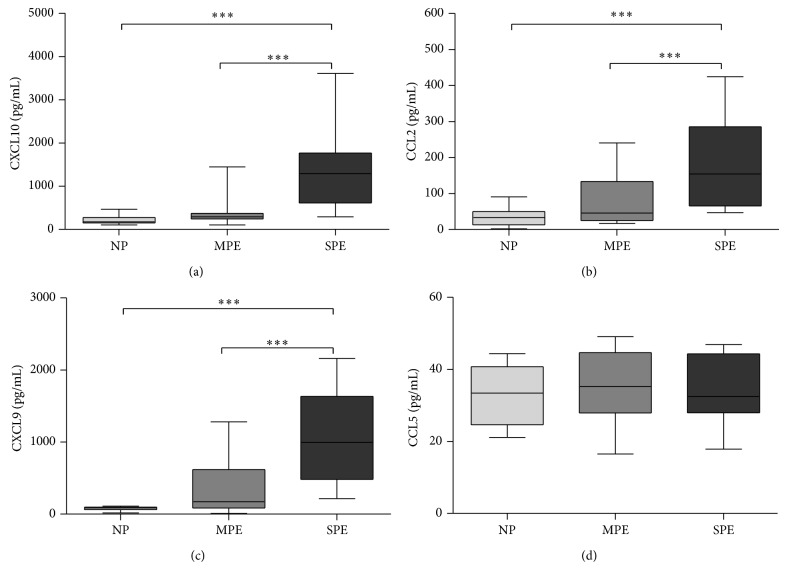
Chemokines related to antiangiogenic properties and inflammatory states are increased in preeclamptic patients. Plasma levels of inflammatory mediators are elevated in preeclamptic women. Plasma was obtained from normotensive pregnant (*n* = 51), moderated preeclamptic pregnant (*n* = 49), and severe preeclamptic pregnant (*n* = 24) women and inflammatory markers were analyzed by CBA microarray (^*^ *P* < 0.05, ^**^ *P* < 0.01, ^***^ *P* < 0.001).

A number of studies have shown that the correct balance between angiogenic and antiangiogenic mediators is critical for the pathogenesis of PE [30]. We detected higher concentrations of CXCL10 (*P* < 0.001) and CXCL9 (*P* < 0.001) in serum from patients with SPE compared with those in MPE and NP (Figures 3(a) and 3(c)).

CXCL10 and CXCL9 are CXC inflammatory chemokines that also act as positive modulators of pathogenesis. The levels of these chemokines are important in PE due to the inadequate blood vessel remodeling. Additionally, these CXC chemokines are secreted in an IFN-*γ*-induced Th1 cytokine environment.

The analyzed SPE patients also presented elevated concentrations of CCL2 in serum compared with the NP (*P* < 0.001) and MPE (*P* < 0.001) patients. These data are also a marker of the systemic inflammatory state in PE (Figure 3(b)).

Interestingly, we did not observe differences in the circulating concentrations of CCL5 between the different patient groups (Figure 3(d)), despite evidence of preeclamptic associations with this CCL chemokine in previous studies.

## 4. Discussion

During pregnancy, immune cells, as T_regs_ cells, that are present in the decidua have a crucial role in maintaining overall homeostasis but have also been implicated in complications such as PE [8, 13]. In the present study, we observed reduced levels of conventional T_regs_ (CD4+CD25^high^Foxp3+ and CD4+Foxp3+) cells in MPE and SPE patients (Figure 1). These data, which correlate with those obtained in previous studies, indicate that T_regs_ subsets are decreased in preeclamptic patients and play key roles in maintaining tolerance [19, 20, 31].

Some studies have suggested that, during pregnancy, T cell responses are reduced by several mechanisms, including the generation of naturally occurring T_regs_, which normally comprise 5–10% of peripheral CD4+ T cells, or induced T_regs_ expressing specific paternal antigens in the mother [8, 13]. Our data suggest that the changes in the levels between T_regs_ subsets in Mexican patients may play a role in the pathogenesis of PE, as previously described by other groups, that suggested that alloantigen-induced T_regs_ (CD4+CD25^high^Foxp3+) may suppress antigens from the fetus.

More recent studies have linked inflammation and a loss of maternal tolerance with PE. Likewise, many experimental works have implicated inflammation with hypertension, which is one of the principal causes of PE complications. Additionally, alterations in the immune response have been implicated in hypertension. For instance, neural T cell activation is increased in hypertension, and athymic mice present hypertension induced by mineralocorticoids [9, 32, 33]. Although T cell response counters the lack of immune tolerance during PE pregnancy complications, the maternal systemic inflammation is activated by the innate immune system, which leads to neutrophil, monocyte, and NK cell activation.

Regarding innate inflammation, our data showed that patients with SPE had elevated circulating concentrations of cytokines compared with MPE and NP patients. In particular, SPE patients had elevated levels of IL-6 in serum, a cytokine that is widely implicated in systemic inflammation and associated with hypertension [34]; in fact, several animal models have shown that IL-6 is one of the mediators of hypertension under hypoxic conditions in the placenta [33, 35].

The TNF-*α* concentrations were elevated in serum in SPE patients, but not in NP and MPE patients. This cytokine has been directly correlated to hypertension because the activation of the AT1 receptor induces TNF-*α* secretion; it is also well known that this cytokine is cytotoxic to villous trophoblast cultures. Additionally, a TNF-*α* soluble receptor attenuates preeclamptic-like features in pregnant rats [9, 36].

The circulating protein levels of other proinflammatory cytokines, such as IL-8, were also significantly elevated in SPE patients. This finding contradicts some works, which have demonstrated that, in placental tissue from patients with PE, IL-8 is produced at lower concentrations, whereas in normal pregnancies, IL-8 production is related to angiogenic activity [37]. However, our data showed that a few SPE patients presented elevated IL-8 protein concentrations, as observed by other groups in maternal and fetus sera from PE patients [38].

Regarding IL-10, no differences between the levels of this cytokine were observed among the patient groups. IL-10 is mainly responsible for the suppression of inflammation. As reported in previous studies, we observed reduced numbers of T_regs_ in SPE patients, but with different systemic concentrations of IL-10. A few papers reported lower IL-10 expression in PE patients compared with healthy pregnant women. IL-10 is also produced by Th2 cells, which is reduced during an immune response in PE [37, 38].

In this paper, we also evaluated the circulating levels of chemokines from PE, finding that CXCL9 and CXCL10 were elevated in SPE patients. It is well known that these chemokines possess antiangiogenic properties, but they are also linked with a Th1-type response because their genes are induced by IFN-*γ* [39]. Additionally, the most important chemoattractant expressed by macrophages CCL2 was elevated in SPE patients. A number of hypotheses have been postulated to explain the onset of placental dysfunction during PE, including generalized endothelial dysfunction, inadequate trophoblast invasion at the fetoplacental junction, and inappropriate maternal inflammatory responses in the placenta [1, 40]. Whether inappropriate inflammatory responses are a primary or secondary cause of preeclampsia remains to be determined.

## 5. Conclusion

Our findings show that the T_regs_ levels are reduced in patients with PE and that the systemic inflammatory status is linked with a severe PE state.

Increased concentrations of IL-6, TNF-*α*, and IL-8 were found in Mexican women at different preeclamptic stages relative to NP patients; no differences in the IL-1*β* and IL-10 levels were detected. An analysis of chemokines in serum also showed higher concentrations of CCL2, CXCL9, and CXCL10 in preeclamptic samples.

## Figures and Tables

**Table 1:** Clinical characteristics of patients with preeclampsia, normotensive pregnant, and nonpregnant healthy women.

	Nonpregnant healthy women (*n* = 27)	Moderated preeclamptic (*n* = 49)	Severe preeclamptic (*n* = 24)	Normotensive pregnant (*n* = 51)
Age (years) (means ± SD)	22.7 ± 2.53	24.9 ± 7.21	23.78 ± 5.68	26.04 ± 7.37
Parity				
Primiparous	2 (7.4%)	26 (53%)	13 (59%)	21 (41%)
Multiparous	1 (3.7%)	23 (47%)	11 (41%)	30 (59%)
Blood pressure (mmHg)				
Systolic	110.25 ± 15.15	158.2 ± 16.75^*^	175.3 ± 11.28^*^	110.3 ± 18.15
Diastolic	73.64 ± 7.11	101.4 ± 5.31^*^	119.3 ± 6.22^*^	71.5 ± 6.32
Gestational age at sampling (weeks)	NA	38.5 (30–41)	35.8 (30–40)	38.4 (30–41)
Gestational age at delivery (weeks) (median, range)	NA	38.5 (30–41)	35.8^*^ (30–40)	39.4 (35–41)
Infant weight (grams) (mean, range)	NA	2 610.8 (560–3 570)	1 617.9^*^ (460–3 110)	2 888.49 (780–3 850)

^*^ *P* < 0.05 preeclamptic patients versus normotensive pregnant women.

NA, not applicable.

## Abbreviations

PE: Preeclampsia
T_regs_: Regulatory T cells
NP: Normotensive pregnant
HC: Healthy controls
MPE: Moderated preeclamptic patients
SPE: Severe preeclamptic patients.
